# Effect of timing of intubation on clinical outcomes of critically ill patients with COVID-19: a systematic review and meta-analysis of non-randomized cohort studies

**DOI:** 10.1186/s13054-021-03540-6

**Published:** 2021-03-25

**Authors:** Eleni Papoutsi, Vassilis G. Giannakoulis, Eleni Xourgia, Christina Routsi, Anastasia Kotanidou, Ilias I. Siempos

**Affiliations:** 1 First Department of Critical Care Medicine and Pulmonary Services, Evangelismos Hospital, National and Kapodistrian University of Athens Medical School, 45-47 Ipsilantou Street, 10676 Athens, Greece; 2 Division of Pulmonary and Critical Care Medicine, Department of Medicine, Weill Cornell Medicine, New York-Presbyterian Hospital-Weill Cornell Medical Center, New York, NY USA

**Keywords:** Coronavirus, Delayed, Pneumonia, Intensive care unit, Acute respiratory failure, Acute respiratory distress syndrome

## Abstract

**Background:**

Although several international guidelines recommend early over late intubation of patients with severe coronavirus disease 2019 (COVID-19), this issue is still controversial. We aimed to investigate the effect (if any) of timing of intubation on clinical outcomes of critically ill patients with COVID-19 by carrying out a systematic review and meta-analysis.

**Methods:**

PubMed and Scopus were systematically searched, while references and preprint servers were explored, for relevant articles up to December 26, 2020, to identify studies which reported on mortality and/or morbidity of patients with COVID-19 undergoing early versus late intubation. “Early” was defined as intubation within 24 h from intensive care unit (ICU) admission, while “late” as intubation at any time after 24 h of ICU admission. All-cause mortality and duration of mechanical ventilation (MV) were the primary outcomes of the meta-analysis. Pooled risk ratio (RR), pooled mean difference (MD) and 95% confidence intervals (CI) were calculated using a random effects model. The meta-analysis was registered with PROSPERO (CRD42020222147).

**Results:**

A total of 12 studies, involving 8944 critically ill patients with COVID-19, were included. There was no statistically detectable difference on all-cause mortality between patients undergoing early versus late intubation (3981 deaths; 45.4% versus 39.1%; RR 1.07, 95% CI 0.99–1.15, *p* = 0.08). This was also the case for duration of MV (1892 patients; MD − 0.58 days, 95% CI − 3.06 to 1.89 days, *p* = 0.65). In a sensitivity analysis using an alternate definition of early/late intubation, intubation without versus with a prior trial of high-flow nasal cannula or noninvasive mechanical ventilation was still not associated with a statistically detectable difference on all-cause mortality (1128 deaths; 48.9% versus 42.5%; RR 1.11, 95% CI 0.99–1.25, p = 0.08).

**Conclusions:**

The synthesized evidence suggests that timing of intubation may have no effect on mortality and morbidity of critically ill patients with COVID-19. These results might justify a wait-and-see approach, which may lead to fewer intubations. Relevant guidelines may therefore need to be updated.

**Supplementary Information:**

The online version contains supplementary material available at 10.1186/s13054-021-03540-6.

## Background

Back to the poliomyelitis epidemic of 1952, Dr. Henry Lassen from Copenhagen had reportedly noted that his American colleagues used to “put their patients in the respirators far too early” [1]. Therefore, since the birth of critical care, the optimal timing of intubation of patients with severe acute hypoxemic respiratory failure seems to be a matter of debate. This debate is reinforced during the ongoing epidemic of coronavirus disease 2019 (COVID-19).

Since the early phase of the COVID-19 epidemic, several guidelines from China [2], United Kingdom [3], United States of America [4] and Australia [5] recommend early intubation of critically ill patients with COVID-19 as a means to protect health care workers from cross-infection and to avoid complications (including cardiac arrest) associated with “crash” intubations. Experts of clinical respiratory physiology seemed to back this approach with notions that early intubation might prevent ensuing patient self-inflicted lung injury [6]. However, on the basis of physiological principles [7], other experts argued against early intubation [8]. Therefore, there seemed to be reasonable arguments in favor of either an early or a late intubation approach in COVID-19 and relevant studies were subsequently planned to address this clinical question.

Several studies reporting on outcomes of patients with COVID-19 undergoing early versus late intubation have indeed been published. However, the accumulated evidence has not yet been synthesized. Thus, we carried out a systematic review and meta-analysis in an attempt to investigate the effect (if any) of timing of intubation on clinical outcomes of critically ill patients with COVID-19.

## Methods

We reported the current systematic review and meta-analysis in accordance with the Preferred Reporting Items for Systematic Reviews and Meta-Analyses (PRISMA) statement [9]. We prespecified inclusion criteria, methods of data synthesis and outcomes in a protocol registered with PROSPERO (CRD42020222147) and available online [10].

### Eligibility criteria

We considered observational cohort studies, which reported on early versus late intubation of critically ill patients with COVID-19 and presented outcomes on all-cause mortality and/or morbidity. Similar to previous systematic reviews on COVID-19 [11], both peer-reviewed papers and preprints were considered in an attempt to take advantage of all rapidly accumulated information. Case reports and case series involving less than 5 patients were excluded.

### Search strategy

Three authors (EP, VGG and EX) independently conducted the literature search and uploaded their findings in an online file storage service (Google Drive) to cross-check them. We systematically searched PubMed and Scopus. We used Boolean logic to create the search key phrase (characteristics AND (“critically ill” OR “ventilated patients”) AND (“non-rebreather” OR NIV OR high-flow OR “mechanical ventilation”)) OR ((timing OR early OR late OR delayed) AND intubation) AND (coronavirus OR covid-19 OR 2019nCoV OR SARS-Cov-2). We also searched references of initially retrieved articles and explored preprint servers (namely, medRxiv and Research Square). We retrieved all relevant literature from December 1, 2019, up to December 26, 2020, with no language restrictions.

### Data extraction and risk of bias assessment

Two authors (VGG and EP) independently extracted data in a prespecified worksheet and cross-checked their findings. We collected data on author, country, type of study, number of critically ill patients with COVID-19, patients’ clinical characteristics and outcomes. When not directly provided, we calculated data of interest, i.e., by subtracting early from total intubations to calculate late intubations. We contacted authors of original contributions. Six authors provided us with additional information, which was incorporated in the findings of the meta-analysis. Details are provided in the Data Supplement (Additional file 1: Supplementary Table 1).

**Table 1:** Characteristics of individual studies and patient populations

First author	Country	Type of study	Patient population, *N*	Early intubation, *N*	Late intubation, *N*	No intubation, *N*	Prior trial of HFNC or NIV, *N*	Female sex, %	Age, years^a^	SOFA at ICU admission^a^
COVID-ICU Group [20]	France Switzerland Belgium	Multicenter, prospective	4244	2635	741	810	NA	26.0	63.0 [54.0–71.0]	5.0 [3.0–8.0]
Grasselli [21]	Italy	Multicenter, retrospective	3988	2929	164	NA	NA	20.0	NA	NA
Hernandez-Romieu [22]^b^	United States	Multicenter, retrospective	175	133	42	NA	78	44.6	66.0 [56.0–75.0]	9.0 [7.0–12.0]
Karagiannidis^c^ [13]	Germany	Multicenter, retrospective	1727	1318	141	145	141	33.6	71.0 [60.0–79.0]	NA
Lee^d^ [23]	Korea	Multicenter, retrospective	47	23	16	8	NA	40.4	70.0 [63.0–77.0]	3.6 (3.2)
Matta^e^ [24]	United States	Single-center, retrospective	111	76	35	NA	81	46.0	68.0 (11.3)	7.6 (3.2)
Mellado-Artigas [25]	Spain Andorra	Multicenter, prospective	468	312	49	NA	92	30.8	61.7 (11.5)	5.8 (2.4)
Pandya^f^ [26]	United States	Single-center, retrospective	75	37	38	NA	NA	42.7	65.0 (14.3)	NA
Roedl [27]	Germany	Multicenter, retrospective	223	128	39	56	46	27.0	69.0 [58.0–77.5]	5.0 [3.0–9.0]
Saida [18]	Tunisia	Single-center, retrospective	10	4	3	3	1	20.0	51.8 (6.3)	NA
Siempos [14]	Greece	Single-center, retrospective	42	14	18	6	11	19.0	65.0 [58.0–71.0]	4.0 [4.0–6.0]
Zuccon [19]	Italy	Single-center, retrospective	54	25	23	6	23	18.5	NA	NA

*N* = number, SOFA = sequential organ failure assessment, ICU = intensive care unit, NA = not available, HFNC = high flow nasal cannula, NIV = noninvasive mechanical ventilation

^a^Data on age and SOFA are presented as means (SD) or medians [IQR], as provided by the individual studies

^b^In the study by Hernandez-Romieu et al., there were three groups: < 8 h, 8–24 h, > 24 h. The first two groups were combined (hence, the two values for age and SOFA) and considered as “early intubation” group for the purpose of this meta-analysis

^c^In the study by Karagiannidis et al., “early” intubation was defined as “non-invasive ventilation (NIV)-failure and intubation within 24 h or intubation without NIV”

^d^In the study by Lee et al., “early” intubation was defined as “intubation/mechanical ventilation and fulfillment of acute respiratory distress syndrome criteria within 24 h”. Data on duration of mechanical ventilation and length of intensive care unit among survivors (rather than among all included patients) were provided in this study

^e^In the study by Matta et al., “early” intubation was defined as “intubation at admission or within 48 h since the onset of increased oxygen requirements”

^f^In the study by Pandya et al., “early” intubation was defined as “intubation within 1.27 days from presentation (median)”

We assessed the methodological quality of the retrieved observational cohort studies with the Tool to Assess Risk of Bias in Cohort Studies, developed by the CLARITY Group at McMaster University [12]. The tool uses 8 questions, with 4 possible answers in each. Details on the risk of bias assessment are provided in the Data Supplement. Three authors (EP, VGG and EX) independently assessed the risk of bias of included studies. Any disagreements were discussed with the corresponding author (IIS).

### Definitions and outcomes of the meta-analysis

We defined “early” intubation as intubation within 24 h from admission in the intensive care unit (ICU). We defined “late” intubation as intubation at any time after 24 h of ICU admission.

The primary outcomes of the meta-analysis were all-cause mortality and duration of mechanical ventilation (MV). The secondary outcomes were ICU length of stay and need for renal replacement therapy.

### Subgroup analyses

We carried out three pre-specified subgroup analyses of (a) critically ill patients undergoing early versus late or no intubation, because we thought that a late intubation approach may occasionally lead to no intubation; (b) studies with low risk of bias; and (c) studies taking place on low disease burden regions, because we thought that an overwhelmed healthcare system may act as a confounder of the association between timing of intubation and clinical outcomes of patients with COVID-19 [13–15]. As low disease burden regions, we considered countries known for low disease activity during the first wave of the pandemic (namely, Germany, Korea and Greece) [13–15].

### Sensitivity analyses

We performed sensitivity analyses on mortality by excluding each study and recalculating the risk ratio (RR) and by excluding studies which used a time threshold other than 24 h from ICU admission for defining early/late intubation. We also performed a sensitivity analysis by considering an alternate definition of early/late intubation using as criterion a prior trial of high flow nasal cannula (HFNC) or noninvasive mechanical ventilation (NIV). Patients intubated without a prior trial of HFNC/NIV were included in the “early intubation” group. Patients intubated with a prior trial of HFNC/NIV were included in the “late intubation” group. The latter group of patients may be prone to the risk of patient self-inflicted lung injury, consequent to an increased respiratory drive leading to high tidal volumes and transpulmonary pressures [6].

### Statistical analysis

We conducted data synthesis using Review Manager 5.4 (RevMan 5.4) by the Cochrane Collaboration [16]. We expressed pooled dichotomous effect measures as RR with 95% confidence intervals (CI) and pooled continuous effect measures as mean difference (MD) with 95% CI. We transformed continuous values presented as medians to means as instructed by the Cochrane Handbook version 6.1, 2020 [17]. We combined means from two different groups in continuous variables, when necessary, using the formula provided by StatsToDo (www.statstodo.com). We assessed the presence of statistical heterogeneity with *I*^2^, interpreted according to the Cochrane Handbook recommendations; 0–40%: might not be important; 30–60%: may represent moderate heterogeneity; 50–90%: substantial heterogeneity; 75–100%: considerable heterogeneity. Regardless of the measured statistical heterogeneity, we thought that clinical heterogeneity might be present due to variability among included studies regarding clinical practices, patient population characteristics and ICU admission criteria and therefore we conservatively utilized a random effects model [17]. A *p* value less than 0.05 denoted statistical significance.

## Results

**Fig. 1:**
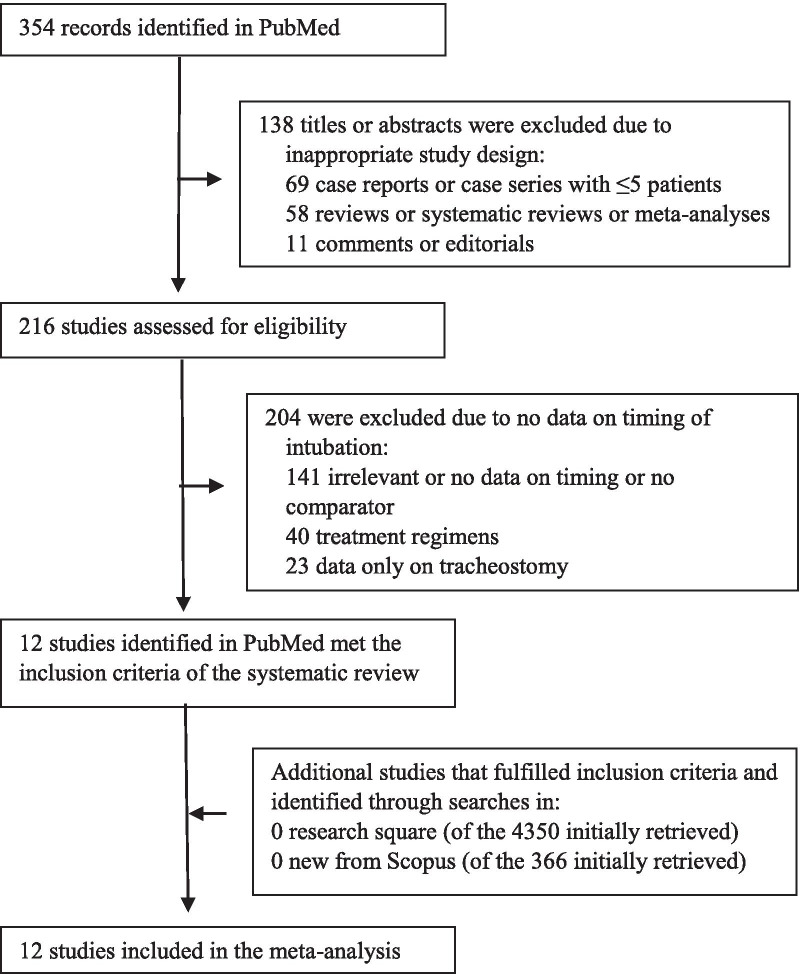
Study flow diagram

Figure 1 shows the flow diagram for study selection. A total of 12 studies [13, 14, 18–27] from Africa, Asia, Europe and America, involving 8944 critically ill patients (7639 early, 1305 late) with COVID-19, were incorporated in our meta-analysis. Table 1 and Additional file 1: Supplementary Table 2 summarize the characteristics and risk of bias assessment of the included studies, respectively.

### Primary outcomes

#### All-cause mortality

**Fig. 2:**
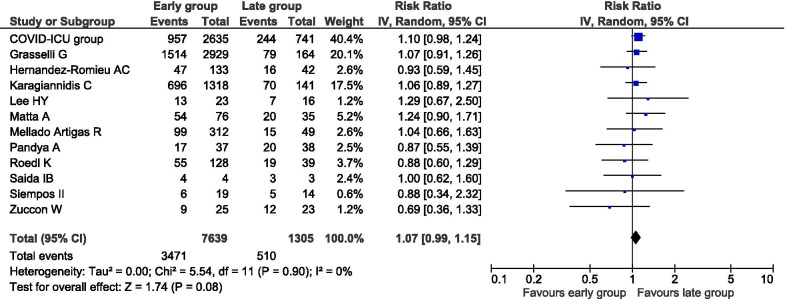
All-cause mortality of patients with COVID-19 undergoing early versus late intubation. Pooled risk ratio (RR) and 95% confidence intervals (CI) were calculated using a random effects model

All 12 studies [13, 14, 18–27] provided data on all-cause mortality. No statistical heterogeneity was detected (*I*^2^ = 0%). There was no statistically detectable difference between patients undergoing early versus late intubation regarding all-cause mortality (3981 deaths; 45.4% versus 39.1%; RR 1.07, 95% CI 0.99–1.15, *p* = 0.08; Fig. 2).

#### Duration of MV

**Fig. 3:**
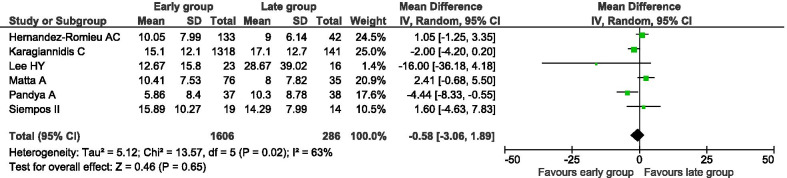
Duration of mechanical ventilation of patients with COVID-19 undergoing early versus late intubation. Mean difference (MD) and 95% confidence intervals (CI) were calculated using a random effects model

Six studies [13, 14, 22–24, 26] provided data on duration of MV. Substantial statistical heterogeneity was observed (*I*^2^ = 63%). There was no statistically detectable difference between patients undergoing early versus late intubation regarding duration of MV (1892 patients; MD − 0.58 days, 95% CI − 3.06 to 1.89 days, *p* = 0.65; Fig. 3).

### Secondary outcomes

#### ICU length of stay

Five studies [14, 22–24, 26] provided data on ICU length of stay. Considerable statistical heterogeneity was detected (*I*^2^ = 78%). There was no statistically detectable difference between patients undergoing early versus late intubation regarding ICU length of stay (433 patients; MD − 1.83 days, 95% CI − 6.05 to 2.38 days, *p* = 0.39).

#### Renal replacement therapy

Five studies [13, 14, 22–24] provided data on renal replacement therapy. No statistical heterogeneity was detected (*I*^2^ = 0%). Need for renal replacement therapy was comparable between early and late intubation groups (547 patients; 30.3% versus 29.0%; RR 1.04, 95% CI 0.83–1.29, p = 0.75).

### Subgroup analyses

In a pre-specified subgroup analysis of eight studies [13, 14, 18–20, 23, 25, 27], all-cause mortality was higher in the early than the late or no intubation group (2377 deaths; 41.2% versus 24.8%; RR 1.54, 95% CI 1.20–1.97, *p* = 0.0007). All-cause mortality was comparable between the early and late intubation group in the subgroup analysis of studies with low risk of bias (four studies [13, 22, 23, 26]; 886 deaths; 51.2% versus 47.7%; RR 1.04, 95% CI 0.89–1.20, *p* = 0.64, *I*^2^ = 0%) and of studies taking place in regions with low disease burden (four studies [13, 14, 23, 27]; 871 deaths; 51.7% versus 48.1%; RR 1.04, 95% CI 0.89–1.21, *p* = 0.63, *I*^2^ = 0%).

### Sensitivity analyses

**Fig. 4:**
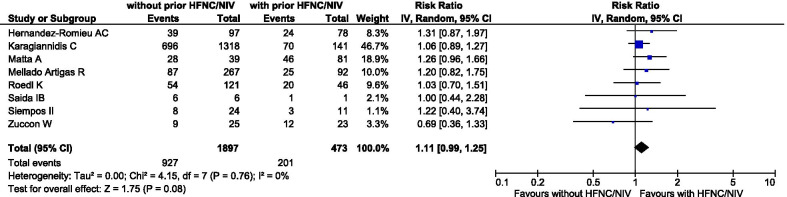
All-cause mortality of patients with COVID-19 undergoing intubation without versus with a prior trial of high flow nasal cannula (HFNC) or noninvasive mechanical ventilation (NIV). Pooled risk ratio (RR) and 95% confidence intervals (CI) were calculated using a random effects model. The authors of two [13, 19] of the studies included in this analysis considered a trial of NIV lasting less than 24 h as inconsequential

After excluding each study and recalculating the RR on mortality, the overall message and statistical significance remained unchanged (Additional file 1: Supplementary Table 3). This was also the case for the analysis after excluding two studies [24, 26] which used a time threshold other than 24 h from ICU admission for defining early/late intubation; i.e., there was no statistically detectable difference between compared groups regarding all-cause mortality (10 studies [13, 14, 18–23, 25, 27]; 3870 deaths; 45.2% versus 38.1%; RR 1.06, 95% CI 0.99–1.15, *p* = 0.11, *I*^2^ = 0%). Finally, in the sensitivity analysis using an alternate definition of early/late intubation, there was no statistically detectable difference on all-cause mortality between patients undergoing intubation without versus with a prior trial of HFNC/NIV (eight studies[13, 14, 18, 19, 22, 24, 25, 27], 1128 deaths; 48.9% versus 42.5%; RR 1.11, 95% CI 0.99–1.25, p = 0.08, *I*^2^ = 0%; Fig. 4).

## Discussion

By incorporating data from 12 studies involving almost 9000 critically ill patients across almost all continents, our systematic review and meta-analysis showed that timing of intubation may have no effect on all-cause mortality, duration of MV, ICU length of stay and renal replacement therapy.

Our systematic review identified variability in the literature regarding the definition of early intubation among the included studies, as presented in Table 1. In an attempt to increase homogeneity, we (inevitably arbitrarily) considered as “early” the intubation occurring within 24 h from ICU admission. We used a specific time threshold from ICU admission as a criterion for defining early/late intubation because this approach has been previously used in the literature both before [28] and during [25] the COVID-19 period. Similarly and interestingly, the Intensive Care National Audit and Research Centre (ICNARC) reports from the United Kingdom provide specific data on patients undergoing intubation within 24 h from ICU admission [29]. Also, given that SARS-Cov-2 virus predominantly affects the respiratory system, one could presume that ICU admission might serve as a surrogate of increased oxygen requirements and subsequent consideration of intubation. Indeed, acute hypoxemic respiratory failure was the main criterion for ICU admission in the largest study included in the meta-analysis [20]. That being said, we acknowledge that there might be variability among centers regarding criteria for ICU admission stemming, for example, from availability of resources, such as beds. In an attempt to address this concern, we used an alternate definition of early/late intubation having a prior trial of HFNC/NIV as criterion.

Regardless of the definition of early/late intubation (i.e., based on a specific time threshold from ICU admission or of a prior trial of HFNC/NIV) used in this meta-analysis, we found no statistically detectable difference on all-cause mortality between patients with severe COVID-19 undergoing early versus late intubation. Also, all-cause mortality was higher in the early than the late or no intubation group. These findings may not support the recommendation in favor of early over late intubation made by several international guidelines [2–5]. Despite the above guidelines, clinicians caring of patients with COVID-19 seem to become eager to favor a wait-and-see strategy over time. Indeed, in a multicentre study from three European countries, involving 4244 critically ill patients with COVID-19, the percentage of patients receiving invasive MV descended from 82 to 68% [16]. A similar trend was reported in a large study from the USA [27]. This shift in intubation strategy from early to late, driven by clinical gestalt, seems to be justified by the findings of this meta-analysis.

We found no statistically detectable difference between patients undergoing early versus late intubation in terms of morbidity, namely duration of MV, ICU length of stay and renal replacement therapy. These findings referring to severe respiratory failure associated with COVID-19 are not in line with findings from observational studies on acute respiratory distress syndrome (ARDS) not associated with COVID-19. The latter studies reported that delaying intubation of critically ill patients with ARDS may be associated with adverse outcomes [28, 30, 31]. This is an interesting observation, which fuels the skepticism regarding the potential differences between ARDS associated with versus without COVID-19 [32, 33].

Our work has limitations. Firstly, as concerns to the outcomes of duration of MV and ICU length of stay, we noted considerable statistical heterogeneity. This probably reflects clinical heterogeneity due to variability among included studies regarding patient population characteristics and clinical practices. Secondly, we did not perform a trial sequential analysis. Thirdly, our meta-analysis is based on data from observational studies, which may suffer from residual confounding. Especially, we could not preclude that confounding by indication may be at play; i.e., sicker patients (with worse prognosis) might get intubated earlier than those with less severe disease. However, we attempted to assess risk of bias of included observational studies and carried out a subgroup analysis of studies with low risk of bias. Fourthly, our subgroup analysis of patients undergoing early versus late or no intubation may be limited by the bias that patients who were not eventually intubated might be, at the first place, less ill than those who were eventually intubated. Finally, the “late intubation” group might include both patients undergoing a time-limited trial of HFNC/NIV and patients remaining on HFNC/NIV for as long as possible. This should be taken into consideration when interpreting the results presented in Fig. 4.

## Conclusions

The synthesized evidence of almost 9000 patients suggests that timing of intubation may have no effect on mortality and morbidity of critically ill patients with COVID-19. These results might justify a wait-and-see approach, which may lead to fewer intubations. Relevant guidelines may therefore need to be updated.

## Supplementary Information

**Additional file1:** Supplementary Tables 1–3 and details on the risk of bias assessment.

## Data Availability

The datasets used and/or analyzed during the current study are available from the corresponding author on reasonable request.

## Abbreviations

COVID-19: Coronavirus disease 2019
ICU: Intensive care unit
MV: Mechanical ventilation
RR: Risk ratio
MD: Mean difference
CI: Confidence intervals
ARDS: Acute respiratory distress syndrome
