# Supplementary material for: Effect of timing of intubation on clinical outcomes of critically ill patients with COVID-19: a systematic review and meta-analysis of non-randomized cohort studies
Source: Crit Care. 2021 Mar 25;25:121. doi: 10.1186/s13054-021-03540-6 (PMC7993905; doi:10.1186/s13054-021-03540-6)
Supplement: Supplementary file 1 — Additional file1: Supplementary Tables 1–3 and details on the risk of bias assessment. [file 13054_2021_3540_MOESM1_ESM.docx]

**Data Supplement**

**Τitle:** Effect of timing of intubation on clinical outcomes of critically ill patients with COVID-19: a systematic review and meta-analysis of non-randomized cohort studies

**Authors:** Eleni Papoutsi, Vassilis G. Giannakoulis, Eleni Xourgia, Christina Routsi, Anastasia Kotanidou, Ilias I. Siempos

**S1. Additional information received after communication with authors of the original studies.**

| **Supplementary Table 1. Additional information provided by authors of original studies.** | | | | |
| --- | --- | --- | --- | --- |
| **Author contacted** | **Data on mortality** | | **Data on a prior trial of HFNC/NIV** | |
|  | **Early intubation**  **(≤ 24 hours)** | **Late intubation**  **(> 24 hours)** | **Intubation without a prior trial of HFNC/NIV** | **Intubation with a prior trial of HFNC/NIV** |
| Alberto Zanella [21] | 1514/2929 | 79/164 | NA | NA |
| Atul Matta [24] | NA | NA | 28/39 | 46/81 |
| Ricard Mellado-Artigas [25] | 99/312 | 15/49 | 87/267 | 25/92 |
| Kevin Roedl [27] | 55/128 | 19/39 | 54/121 | 20/46 |
| Ilias I. Siempos [14] | 6/19 | 5/14 | 8/24 | 3/11 |
| William Zuccon [19] | NA | NA | 9/25 | 12/23 |

*Abbreviations:* HFNC= high flow nasal cannula; NIV= non-invasive mechanical ventilation; NA= not available/not applicable.

Data are presented as “events/total number of patients”.

David A. Harrison and J. Duncan Young informed us that their study does not include relevant data and was not incorporated in our meta-analysis ([Paloma Ferrando-Vivas](https://pubmed.ncbi.nlm.nih.gov/?term=Ferrando-Vivas+P&cauthor_id=33116052) , [James Doidge](https://pubmed.ncbi.nlm.nih.gov/?term=Doidge+J&cauthor_id=33116052) et al. Prognostic Factors for 30-Day Mortality in Critically Ill Patients With Coronavirus Disease 2019: An Observational Cohort Study. *Critical Care Medicine*, 2021) .

**S2. Risk of bias explanation and results**

The following questions are derived from the “Tool to assess risk of bias in cohort studies” contributed by the CLARITY Group at McMaster University:

<https://www.evidencepartners.com/wp-content/uploads/2017/09/Tool-to-Assess-Risk-of-Bias-in-Cohort-Studies.pdf>

The questionnaire divides the cohorts as exposed and non-exposed. The examples beneath the questions are intended to clarify the rationale behind answers in each question.

In each question, 4 answers were possible:

1.Definitely yes (low risk of bias)

2. Probably yes

3. Probably no

4. Definitely no (high risk of bias)

***Q1. Was selection of early and late cohorts drawn from the same population?***

**Definitely yes:** Early and late patient cohorts drawn from same administrative database of patients presenting at same points of care over the same time frame (study period <2 months)

**Probably yes:** Early and late patient cohorts drawn from same administrative database of patients presenting at same points of care over a time frame of 2-6 months

**Probably no:**  Early and late presenting to different points of care (eg multicenter study) or over a time frame of >6 months

**Definitely no:** Early and late presenting to unspecified points of care or over a different time frame (stated or implied shift in intubation strategy during the time period)

***Q2. Can we be confident in the presence of early and late intubation?***

Due to the nature of our cohort’s interventions, **a definitely yes** was prespecified as the appropriate answer

***Q3. Can we be confident that the outcome of interest was not present at start of study?***

All of the outcomes of interest occurred after COVID-19 infection, especially in mortality.

Therefore, a **definitely yes** was prespecified as the appropriate answer .

***Q4. Did the study match exposed and unexposed for all variables that are associated with the outcome of interest or did the statistical analysis adjust for these prognostic variables?***

**Definitely yes:** Matching or adjustment for all the prognostic variables on early/late intubation outcomes

**Probably yes:** Matching or adjustment for some prognostic variables on early/late intubation outcomes

**Probably no:** Matching or adjustment for one prognostic variable on early/late intubation outcomes

**Definitely no:** No matching or adjustments for prognostic variables on early/late intubation outcomes

***Q5. Can we be confident in the assessment of the presence or absence of prognostic factors?***

**Definitely yes:** Data collection on prognostic variables through electronic medical records

**Probably yes:** Data collection through database or review of charts

**Probably no:** Data collection without demonstration of reproducibility

**Definitely no:** Data collection process not stated or no data on prognostic factors regarding early and late groups

***Q6. Can we be confident in the assessment of outcome?***

Due to the nature of our primary outcome, **a definitely yes** was prespecified as the appropriate answer.

***Q7.Was the follow up of cohorts adequate?***

**Definitely yes:** Median follow up of at least 28 days, or all patients discharged or dead

**Probably yes:** Median follow up between 14-28 days

**Probably no:** Median follow-up between 7 and up to but not including 14 days

**Definitely no:** Median follow-up less than 7 days or not stated

***Q8. Were co-Interventions similar between groups?***

Due to the nature of the variability concerning the co-interventions between early and late groups (eg. differences in sedation), **a probably no** was prespecified as the appropriate answer

| **Supplementary Table 2. Risk of bias assessment results.** | | | | | | | | |
| --- | --- | --- | --- | --- | --- | --- | --- | --- |
| **AUTHOR** | **Q1** | **Q2** | **Q3** | **Q4** | **Q5** | **Q6** | **Q7** | **Q8** |
| COVID-ICU Group [20] | DN | DY | DY | DN | DN | DY | DY | PN |
| Grasselli [21] | PN | DY | DY | DN | DN | DY | DY | PN |
| Hernandiez-Romieu [22] | DN | DY | DY | DY | DY | DY | DY | PN |
| Karagiannidis [13] | PN | DY | DY | DN | DY | DY | DY | PN |
| Lee [23] | PN | DY | DY | PY | DY | DY | DY | PN |
| Matta [24] | DN | DY | DY | DN | DN | DY | DY | PN |
| Mellado-Artigas [25] | PN | DY | DY | DN | DN | DY | DY | PN |
| Pandya [26] | PY | DY | DY | DN | DY | DY | PY | PN |
| Roedl [27] | PN | DY | DY | DN | DN | DY | DY | PN |
| Saida [18] | DY | DY | DY | DN | DN | DY | DY | PN |
| Siempos [14] | DY | DY | DY | DN | DN | DY | DY | PN |
| Zuccon [19] | PY | DY | DY | DN | DN | DY | PY | PN |
| Q: Question; DY: Definitely Yes; PY: Probably Yes; PN: Probably No; DN: Definitely No | | | | | | | | |

**S3. Sensitivity analysis by sequential exclusion of each study included in the meta-analysis.**

| **Supplementary Table 3. Sensitivity analysis by sequential exclusion of each study.** | | | |
| --- | --- | --- | --- |
| **Study excluded** | **Risk ratio** | **95% Confidence intervals** | **p-value** |
| COVID-ICU Group [20] | 1.04 | 0.95-1.15 | 0.39 |
| Grasselli [21] | 1.06 | 0.98-1.15 | 0.13 |
| Hernandez-Romieu [22] | 1.07 | 0.99-1.15 | 0.07 |
| Karagiannidis [13] | 1.07 | 0.98-1.16 | 0.11 |
| Lee [23] | 1.06 | 0.99-1.14 | 0.10 |
| Matta [24] | 1.06 | 0.98-1.14 | 0.14 |
| Mellado-Artigas [25] | 1.07 | 0.99-1.15 | 0.08 |
| Pandya [26] | 1.07 | 1.00-1.15 | 0.07 |
| Roedl [27] | 1.07 | 1.00-1.16 | 0.06 |
| Saida [18] | 1.07 | 0.99-1.15 | 0.08 |
| Siempos [14] | 1.07 | 0.99-1.15 | 0.08 |
| Zuccon [19] | 1.07 | 1.00-1.15 | 0.06 |
